# The effect of single dose albendazole (400 mg) treatment on the human gut microbiome of hookworm-infected Ghanaian individuals

**DOI:** 10.1038/s41598-023-38376-3

**Published:** 2023-07-12

**Authors:** Francis Appiah-Twum, Jewelna Akorli, Lydia Okyere, Kate Sagoe, Dickson Osabutey, Michael Cappello, Michael D. Wilson

**Affiliations:** 1 Department of Parasitology, Noguchi Memorial Institute for Medical Research, University of Ghana, PO Box LG 581, Legon, Accra, Ghana; 2 Department of Pathobiology, University of Illinois, Urbana-Champaign, 2522 Vet Med Basic Sciences Bldg., 2001 South Lincoln Avenue, Urbana, IL 61802 USA; 3 Pan African University Institute for Basic Sciences, Technology, and Innovation (PAUSTI), P. O. Box 62000 00200, Nairobi, Kenya; 4 Department of Epidemiology of Microbial Diseases, Yale School of Public Health, Yale University, 60 College St, New Haven, CT 06520 USA

**Keywords:** Metagenomics, Next-generation sequencing, Parasitic infection

## Abstract

Microbes play a key role in human gut homeostasis, metabolic, immunologic and physiopathology of the body. A longitudinal study conducted during 2018–2021 in the Kintampo North Municipality in Ghana demonstrated low hookworm infection cure rates following treatment with a single dose of 400 mg albendazole in some communities. To investigate associations between hookworm infection and the gut microbiome, we examined stool samples from consented participants who were either cured or remained infected after treatment. At each time point, stool was collected prior to and 10–14 days after albendazole treatment. We used 16S rRNA amplicon sequencing of DNA extracted from stool samples to investigate the composition and diversity of the gut microbiota and to identify potential microbial biomarkers associated with treatment outcomes. Hookworm infection was associated with increased species richness (*p* = 0.0093). Among treated individuals, there was also a significant variation in microbiota composition at 10–14 days following single-dose albendazole treatment. Individuals cured of hookworm infection after treatment showed a significant reduction in microbiota composition when compared to their pre-treatment state (ANOSIM; *p* = 0.02), whilst individuals who failed to clear the infection showed no change in microbiota composition (ANOSIM; *p* = 0.35). Uninfected individuals and those who were successfully treated were similar in their microbial composition and structure. We also found that the abundance of *Clostridia* spp. was increased in infected individuals pre- or post-treatment. Predictive functional profiling revealed the enrichment of two pyruvate ferredoxin oxidoreductase subunit pathways in individuals who remained infected after treatment (*p* < 0.05), alluding to an upturn of strictly anaerobic commensal bacteria such as *Clostridia* spp. This study suggests a relationship between human gut microbiome dysbiosis and albendazole therapy outcomes of hookworm infection. Future studies will further characterize specific biomarkers identified within this study to establish their potential for assessment of pharmacological responses to anthelminthic therapies, as well as explore the possibility of using probiotic supplementation as an adjunct treatment to increase albendazole effectiveness against hookworm.

## Introduction

The human gut microbiota is a diverse collection of microbes (i.e., bacteria, fungi, and viruses) that thrive in the gastrointestinal tract of humans^1^. They play an integral role in the competent biological functioning of the human body, due to the complex interplay that exists between enteric bacteria and human cells^2^. This symbiotic relationship allows for proper host metabolic functioning^3^, ensures adequate immune modulation^4^ and provides protection against pathogenic microorganisms, among a host of other functions^5^.

In addition to microbes, other pathological organisms such as helminths can also colonize the human gut^6^. Hookworms, particularly *Necator americanus and Ancylostoma duodenale*, are soil-transmitted helminths (STH) responsible for approximately 472 million human infections worldwide. These infections mainly occur in disadvantaged communities situated in tropical and subtropical regions ^7^. Upon host infection, hookworm larvae are carried from the site of skin penetration through the bloodstream to the lungs, after which the larvae migrate up the trachea to be swallowed. The ingested larvae settle in the lining of the duodenum where they develop to the adult, blood feeding stage ^8^. Scientific evidence suggests that helminth infections dynamically alter the structure and composition of the intestinal microbiota^9^. This results from microbiota sensitivity to homeostatic imbalances within the human gastrointestinal tract^10^, a phenomenon referred to as helminth-induced human gastrointestinal dysbiosis.

Hookworm infections are routinely treated with albendazole (albendazole sulfoxide), a benzimidazole drug that causes degeneration of microtubules within intestinal and tegument cells of adult worms and larvae^11^. The World Health Organization (WHO) recommends preventive chemotherapy for populations at risk of STH infections with the oral administration of a single dose of 400 mg albendazole either annually or biannually^12^. STH resistance to benzimidazoles is well documented in animals such as livestock^13^ but not in humans, partly due to limited monitoring of anthelminthic response in communities subjected to mass drug administration (MDA)^14,15^. The overall state of albendazole efficacy in the treatment of human hookworm infections, therefore, remains unclear. Historically, single-dose albendazole therapy has shown adequate efficacy in the treatment of human hookworm infections with moderately high cure rates^16–18^. However, there are recent reports of reduced cure rates especially in areas where treatment has been extensive due to MDA efforts^19–21^.

The gut microbiome has been shown to play a role in helminth infections, and studies to identify the associations between gut microbes and deworming treatment outcomes have gained interest^22^. The current study aimed to investigate the influence of single-dose 400 mg albendazole on the human gut microbiota of individuals in communities with reported low cure rates to gain deeper insights into the relationship between gut microbial alteration and reduced responsiveness to therapy.

## Materials and methods

### Study design

**Figure 1:**
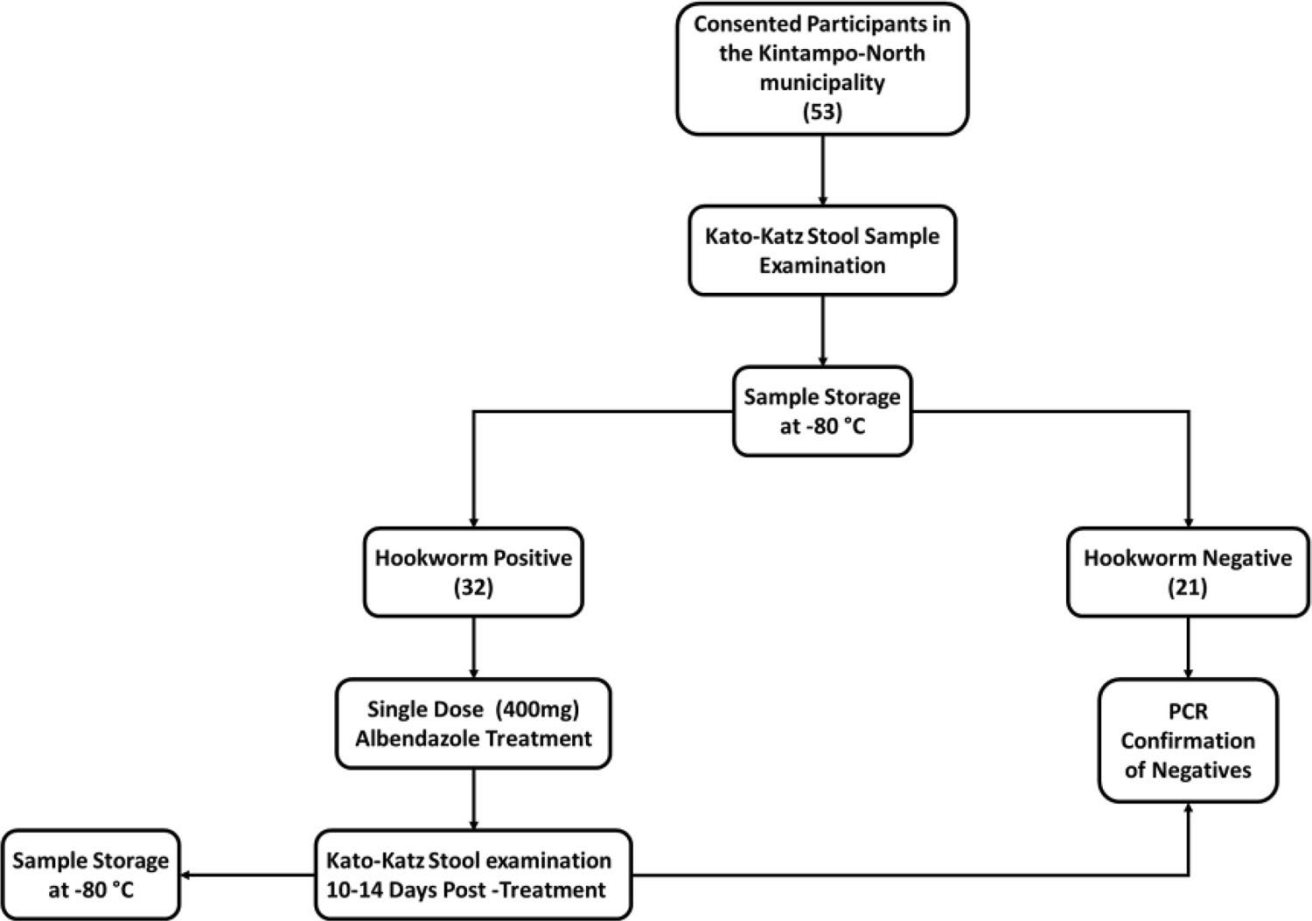
Sample collection, processing, and storage at baseline.

This study was a nested case–control study from a larger cohort study under the NIH/NIAID-funded Tropical Medicine Research Centre (TMRC), the Noguchi Memorial Institute Initiative for NTDs Elimination (NIINE). For the NIINE study, stool samples were collected from consented individuals over a two-year period in the Kintampo North Municipality, Ghana. At each sample collection event i.e., baseline, 9 and 18 months, stool samples were prepared using the Kato-Katz technique^23^ and examined for the presence of hookworm eggs with the help of a compound microscope at 10 × and 40 × objectives. Each slide was inspected independently by two experienced technologists, and a third for quality control. The remaining stool samples were stored at − 80 °C within 6 h of collection (Fig. 1).

Hookworm-positive participants were treated with a single dose of 400 mg albendazole within 24 h of the first stool collection, and the treatment outcome was assessed 10–14 days after by examining stool samples as described. Those who remained infected were categorized as ‘treatment failures. The hookworm infection status of all microscopy-negative samples was further corroborated using polymerase chain reaction amplification (PCR) method for the identification of both *N. americanus* and *A. duodenale*^24^.

In this microbiome-related study, 97 stool samples were obtained from 53 individuals selected from archived samples. The sample selection was based on infection status and treatment outcomes. Positive samples were selected based of Kato-Katz results whilst only PCR confirmed negative samples were selected as true negatives. The availability of samples and complete stool data for both pre-treatment and post-treatment was also taken into consideration during sample selection. (Table 1).

**Table 1:** Microbiome study sample groups, descriptions, and respective sample sizes.

Treatment outcomes	Group	Group description	Sample size
Successful therapy (ST)	Pre-ST	Baseline positive samples of successfully treated individuals	16
	Post-ST	Post-treatment samples of successfully treated individuals	16
Failed therapy (FT)	Pre-FT	Baseline positive samples of individuals that failed treatment	11
	Post-FT	Post-treatment samples of individuals that failed treatment	11
Successive reinfection	SR	Samples from individuals that were reinfected post successful treatment at multiple timepoints	16
Constant therapy failure	CTF	Samples from individual that remained positive at all timepoints	6
No treatment controls	NTC	Baseline negative samples	21

### 16S rRNA gene amplicon sequencing

Genomic DNA was extracted using a modified extraction protocol of the Quick-DNA Faecal/Soil Microbe DNA Miniprep Kit from Zymo Research. The samples were lysed mechanically via bead beating using a high speed vortexing and chemically using lysis buffers to ensure adequate debris elimination and maximise DNA yield. To control for potential contamination in downstream analyses, two mock samples (no-template controls) were included during the extraction process. The concentrations of all DNA samples were determined with a Qubit Fluorometer 2.0 (Invitrogen).

The V3–V4 hypervariable region of the bacterial 16S rRNA gene was amplified by PCR using 341F (5′-CCTAYGGGRBGCASCAG-3′) and 806R (5′-GGACTACNNGGGTATCTAAT-3′) primers. A 16S rRNA sequencing library was prepared according to the 16S Metagenomic Sequencing Library Preparation protocol (Illumina™, Inc., San Diego, CA, United States)^25^. Samples were multiplexed and individual barcode sequences were added to each DNA fragment during next-generation sequencing (NGS) library preparation and, sequenced on the NovaSeq 6000 Illumina platform following standard Illumina sequencing protocols.

### Sequence filtering and taxonomic annotation

Paired-end raw reads of length 250 bp and were generated per sample after sequencing. Sequence filtering, clustering and taxonomic classifications were performed with established plugins in QIIME2 version 2020.8.0^26^. Denoising, chimeric read detection and removal were performed based on read quality^23^ using the *dada2 denoise-paired* command^26,27^. *Dada2* performed the read merging automatically after denoising resulting in distinct Amplicon Sequence Variants **(**ASVs) samples^25^.

Operational Taxonomic Unit (OTU) clustering was achieved with closed-reference method using the *vsearch* plugin^26,28^. ASVs with ≥ 97% sequence similarity in the SILVA SSU rRNA database 138, were assigned to the same Operational Taxonomic Unit (OTU). Taxonomic annotation of OTUs to the species level was performed against the SILVA SSU rRNA database 138 to a confidence threshold of 0.8–1. Phylogenetic relationships between OTUs were established following multiple sequence alignments using the *phylogeny* plugin through the *align-to-tree-mafft-fasttree* command^26^.

### Contaminant removal and data normalization

To control for biological contaminants, two mock samples (no-template controls) were processed together with the study samples. The sequence data of the mock samples were used to identify and remove ‘contaminant’ sequences in the test samples at sequence similarity threshold of 50%. This process was performed using the *decontam* package in R (version 4.1.3)^29,30^.

To enable accurate comparison of downstream statistics for different computational measurements, the clustered OTUs were normalized using rarefaction. Samples were rarefied to a depth equal to the minimum sequencing depth within the dataset. The rarefaction was executed using the *rarefy_even_depth* function of the *Phyloseq* package in R (version 4.1.3)^29,31^.

### Diversity analyses

Species richness and, Shannon Entropy and Gini-Simpson alpha-diversity indices, which account for effective number of species, were calculated to determine the complexity of sample biodiversity among samples within the same group. This was performed using the *get_alphaindex* function in the *MicrobiotaProcess* package in R (version 4.1.3)^29–32^.

Beta diversity was investigated with Permutational multivariate analysis of variance (PERMANOVA) and Analysis of similarity (ANOSIM) using the *adonis* and *anosim* functions respectively on weighted Unifrac distances in the R *vegan* package^29,33^. These methods were used to test for the significance of structural and compositional differences between sample group microbial communities to determine the existence of significant dissimilarities across sample groups. Non-metric multi-dimensional scaling (NMDS) was used to graphically represent these between-group dissimilarities.

### Microbial biomarker discovery

Wilcoxon rank-sum test and Kruskal Wallis test were performed using linear discriminant analysis (LDA) to establish the association between the taxonomic abundance of individual microbial taxa within sample groups based on treatment outcomes. These tests were executed using the *diff_analysis* function in the *MicrobiotaProcess* package in R (version 4.1.3)^29,31^. A microbial taxon is considered a potential biomarker if it had an LDA score ≥ 4 and, *p*-value ≤ 0.05 and ≤ 0.01 after Wilcoxon and Kruskal Wallis tests, respectively.

### Predictive functional profiling

The KEGG Orthologs (KO)^34^ were generated using the *full pipeline.py* command of PICRUSt2 (version 2.4.1)^35^. This was done to predict the abundance of key high-level pathways within sample group microbial communities using the OTUs obtained from 16S rRNA gene sequencing. KO abundances within sample groups were visualized using the STAMP software^36^.

### Ethical approval

This study received ethical approvals from the Noguchi Memorial Institute for Medical Research (NMIMR/CPN#: 100/16-17), NIAID DMID (# 17-0061), Kintampo Health Research Centre (KHRCIEC2017-20) and Council for Scientific and Industrial Research (RPN 008/CSIR-IRB/2017). Written informed consent was obtained directly from all adult participants and legal guardians provided informed consent on behalf of all minors (below 18 years) within the study group. Procedures in this study were performed in accordance with the Ghana Public Health Act, 2012 (Act 851) and the Data Protection Act, 2012.

## Results

### Removal of contaminant sequences

A total of 4153 unique sequences were obtained after 16s rRNA sequencing. 147 sequences of which were purged after contaminant removal representing 3.5% of unique sequences. As such, 4006 unique Operational Taxonomic Units (OTUs) were identified and adequately depicted the total microbiota composition.

### Assessment of microbiome pre-treatment

**Figure 2:**
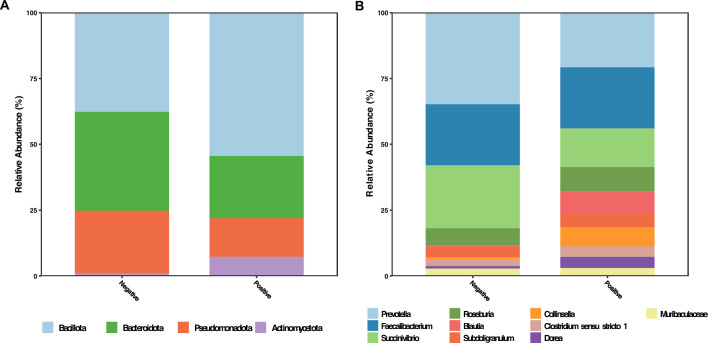
Taxonomic distribution of pre-treatment hookworm positive and negative samples with relative abundances greater than 1%. (**A**) Phylum. level. (**B**) Genus level.

We used a cross-sectional approach to first compare the microbiome of 32 hookworm positives and 21 negative individuals. Taxa with average relative abundance greater than 1% were compared between the sample groups. The hookworm positive sample group showed a higher relative abundance of both *Bacillota* (*p* = 0.0028). and *Actinomycetota* (*p* < 0.0001). compared to uninfected individuals, while *Bacteroidota* (*p* = 0.0058). and *Pseudomonadota* (*p* = 0.04). were less abundant (Fig. 2A).

Abundance distribution at the genus level revealed that *Prevotella, Succinivibrio* and *Collinsella*, accounted for the greatest proportion of taxa within the *Bacteroidota*, *Pseudomonadota* and *Actinomycetota* phyla, respectively. *Bacillota* on the other hand demonstrated a wider distribution, with six genera having relative abundances greater than 1%. Among these genera *Blautia*, *Subdoligranulum*, *Dorea* and *Clostridium *sensu stricto* 1* had increased relative abundances in the positive group (Fig. 2B).

**Figure 3:**
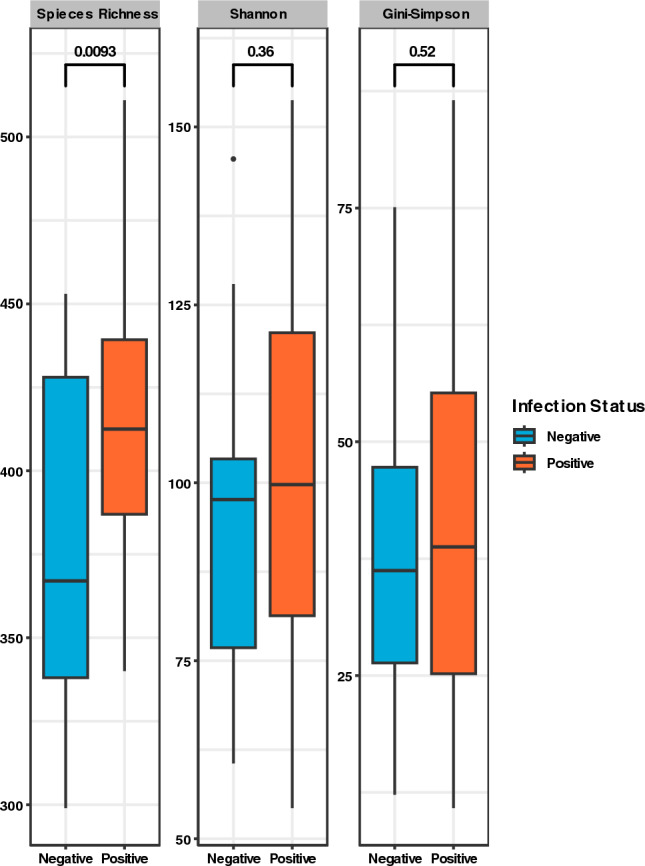
Alpha diversity plot comparing microbial diversity among negative (blue) and positive (orange) individuals pre-treatment. Both Shannon and Gini-Simpson indices were calculated to represent effective number of species.

Positive samples recorded higher median values across all alpha diversity indices estimated (Fig. 3). The variances in diversity within the sample groups, however, did not differ i.e., *p-*values for Shannon and Simpson > 0.05, although species richness showed a significant difference between positive and negative samples (*p* = 0.0093).

**Figure 4:**
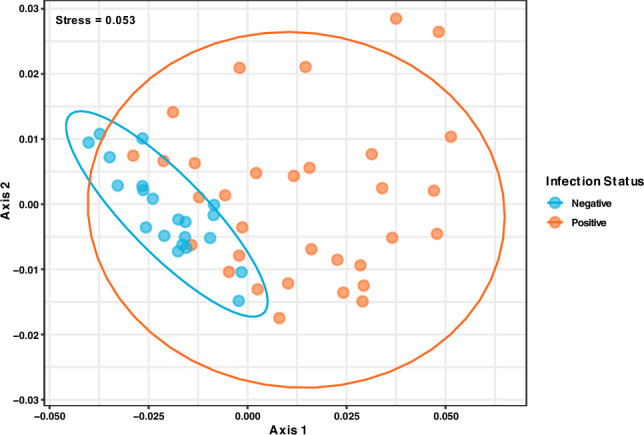
Non-metric multidimensional scaling (NMDS) plot comparing microbiome structure across negative (blue) and positive (orange) individuals pre-treatment.

Comparing the microbial diversity between hookworm infected and uninfected individuals revealed substantial dissimilarity (PERMANOVA: *p* = 0.007*,* R^2^ = 0.063). It was observed with non-metric multidimensional scaling (stress = 0.053) that both groups formed distinct clusters implying differences in microbiome structure and reiterating high variance in positive hookworm individuals (Fig. 4).

**Figure 5:**
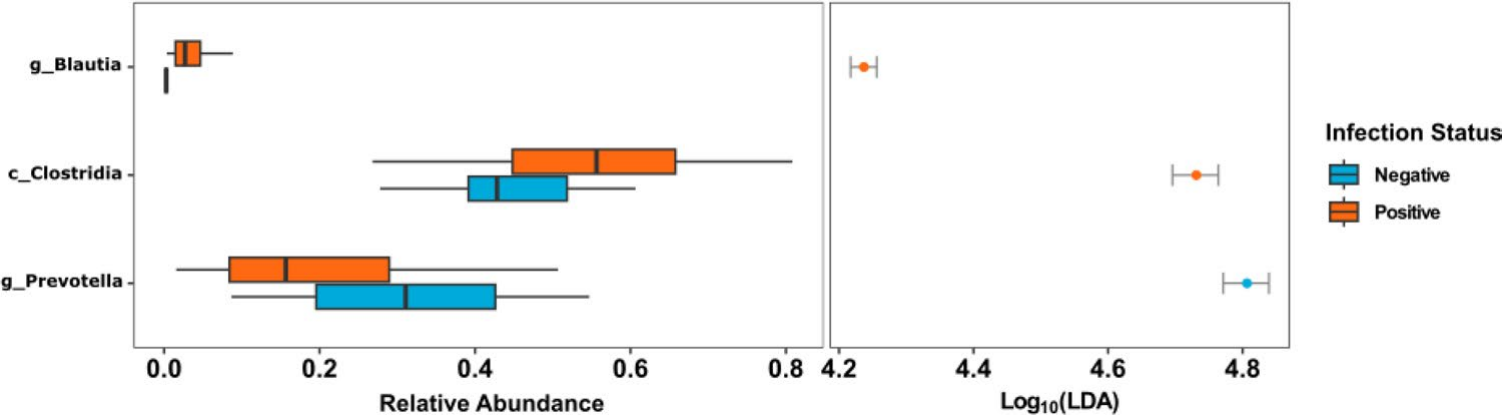
LDA plot outlining significantly associated microbial taxa within positive (orange) and negative (blue) samples before treatment.

Employing linear discriminant analysis (LDA), deferential microbial testing was performed to identify potential microbial taxa driving dissimilarities between the infection groups. When baseline positive and negative samples were compared, positive samples showed a significantly higher relative abundance of *Clostridia* and *Blautia* while *Prevotella* was significantly higher within negative samples (LDA score > 4.00) (Fig. 5).

### Effect of albendazole treatment on gut microbiome

The microbiomes of individuals were assessed in stool samples collected before and 10–14 days after treatment to provide insight into the effects of deworming. Pre- and post-treatment samples for successful therapy (pre-ST vs post-ST) and the failed therapy (pre-FT vs post-FT) groups were compared separately to observe how the microbiome is altered after either successful or sub-optimal treatment. Microbiota abundance was reduced after drug administration in both successful therapy and failed therapy groups. However, this reduction was only significant after successful clearance (PERMANOVA: *p* = 0.01, R^2^ = 0.10873) and not with the failed therapy group (PERMANOVA: *p* = 0.78, R^2^ = 0.02024).

**Figure 6:**
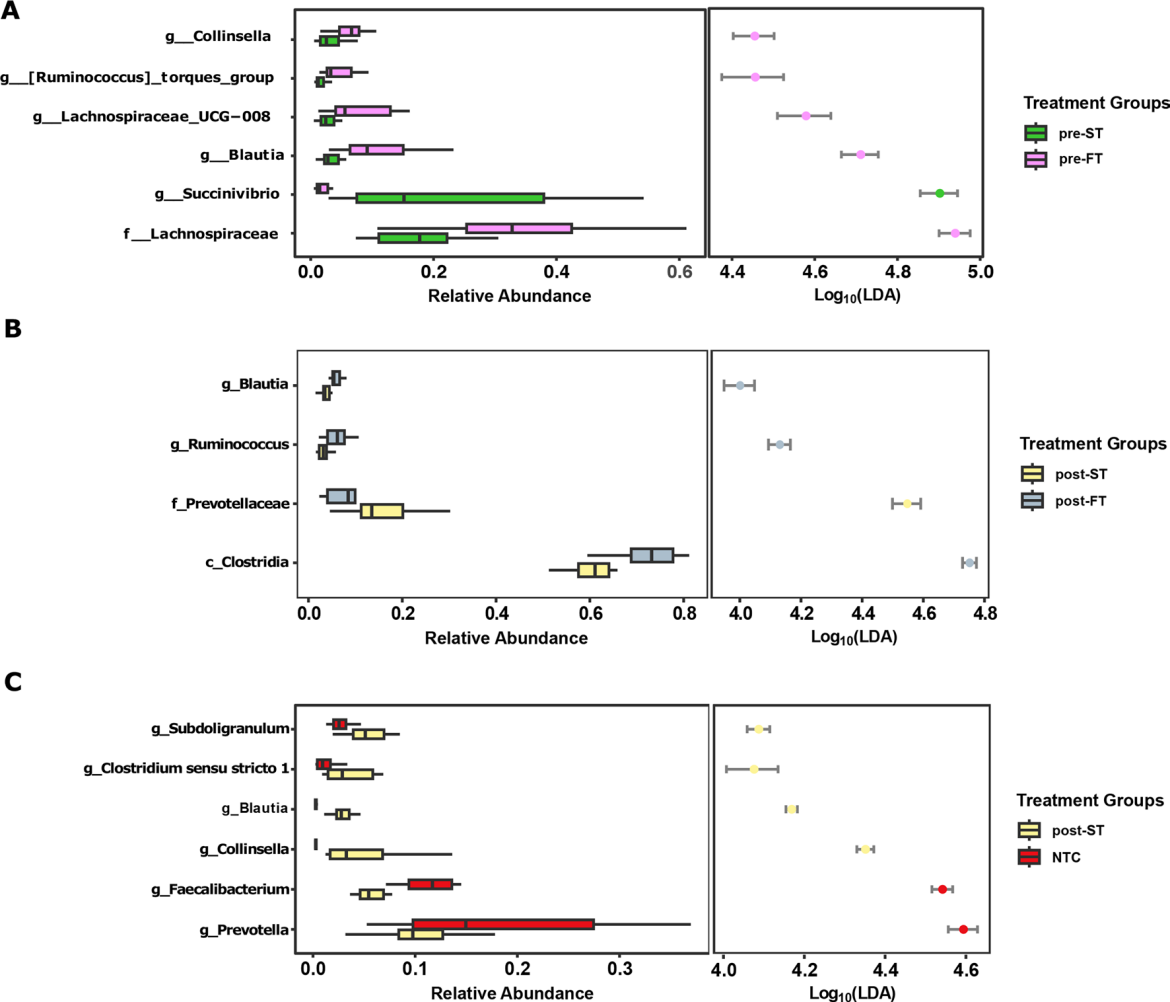
LDA plots outlining significantly associated microbial taxa based on treatment outcome. (**A**) Comparison between successful infection clearance (green) and treatment failure samples (purple) before treatment. (**B**) Comparison between successful infection clearance (yellow) and treatment failure samples (grey) after treatment. (**C**) Comparison between infection clearance (yellow) and no infection controls (red).

We further compared the microbiome structure and composition of individuals before and after treatment i.e., those that cleared infection (ST) against individuals who remained infected (FT) after treatment. Before and after treatment the microbiota composition of FT samples was represented most significantly by taxa within the class *Clostridia* such as *Lachnospiraceae, Blautia* and *Ruminococcus*. ST samples, on the other hand were represented by an increased abundance of other taxa such as *Succinivibrio* and *Prevotellaceae* (LDA score > 4) (Fig. 6A,B).

As part of our study, we also sought to investigate whether clearing the hookworm infection led to significant restructuring of the gut microbiome to a non-infected state. However, we noticed that even after complete clearance of the helminth infection, a significant difference in the microbiota composition existed between the two groups, implying the microbiome did not revert to a non-infected state (PERMANOVA: *p* = 0.0004, R^2^ = 0.22612). When compared to baseline negative samples (NTC), individuals cleared after treatment had a higher abundance of *Collinsella and Clostridia* spp*.* such as *Subdoligranulum*, *Clostridium_sensu_stricto_1* and *Blautia* (Fig. 6C).

### Predictive function of gut microbiome in successful and failed treatment groups

**Figure 7:**
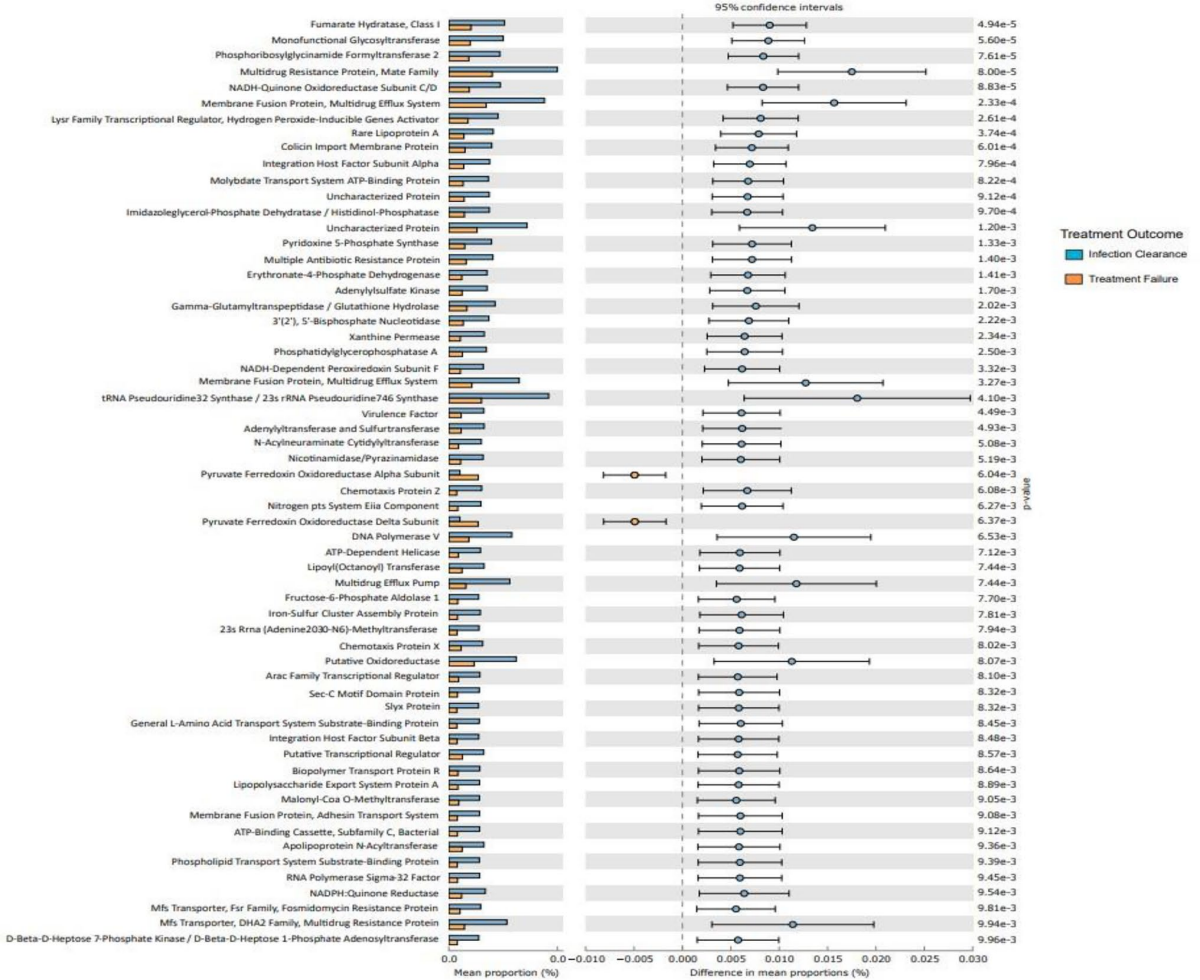
Predictive functional analysis outlining high-level KEGG pathways^34^ associated with infection clearance (blue) and failed treatment (orange) groups.

We also investigated microbiota function based on predicted metagenomes, and compared post-ST and post-FT in the Kyoto Encyclopaedia of Genes and Genomes (KEGG) orthology (KO). Among 559 affiliated KEGG pathways, 65 differed between post-ST and post-FT groups (*p* < 0.05). Among these, 63 pathways were associated with the successful treatment group and 2 with the failed treatment group (*p* < 0.05) (Fig. 7).

Interestingly, pathways associated with metabolism, biosynthesis of cofactors and membrane transporters were enriched in the successful treatment group whilst the failed treatment group showed enrichment of pyruvate ferredoxin oxidoreductase (PFOR) subunit pathways.

### Gut microbiome, chronicity and post-treatment reinfection

Individuals who demonstrated a pattern of reinfection (SR) were also analysed for association of microbial biomarkers during each time-point. These individuals included those who presented with hookworm at baseline, cleared the infection following treatment but were infected again at the 9-month follow-up study. There was no variation in microbial diversity across samples within the successive reinfection group (PERMANOVA: *p* = 0.17, R^2^ = 0.02024), suggesting that the treatment may have resulted in an incomplete clearing leading to undetectable low levels of infections which became observable in the 9-month follow-up.

**Figure 8:**
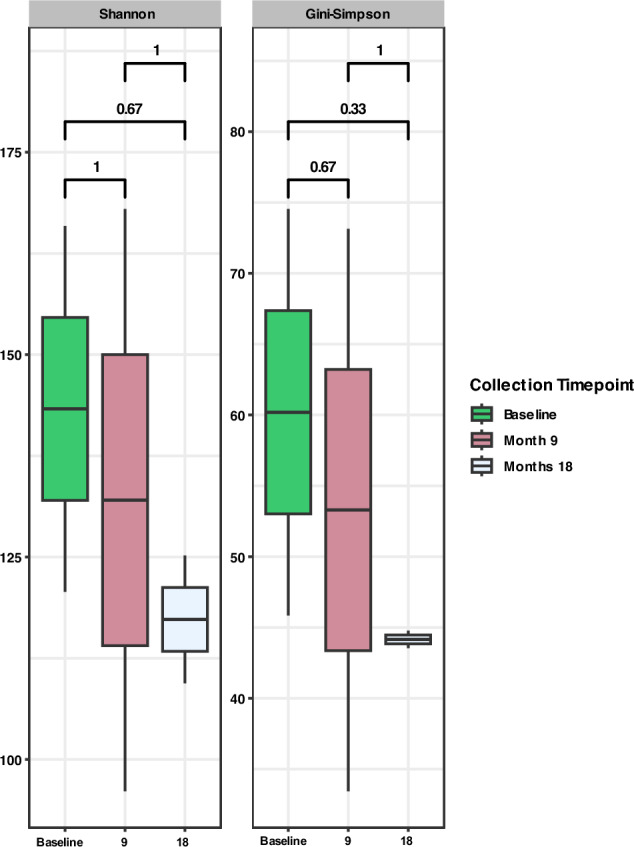
Alpha diversity plot comparing microbial diversity of CTF across all sample collection timepoints; baseline (green), 9 months (pink) and 18 months (grey). Both Shannon and Gini-Simpson indices were calculated to represent effective number of species.

Across the larger cohort, one individual maintained a persistent hookworm infection over all sample collection events, designated hereafter as ‘consistent treatment failure’ (CTF). The microbiome state of this individual was also analysed to assess the effects of progressive treatment failure and chronicity of hookworm infection. Interestingly, the effective number of species within the microbiome of the individual reduced at every timepoint (i.e., baseline, 9 and 18 months), although not statistically significant (Fig. 8).

## Discussion

The physiological tug of war between parasitic helminths and gut microbiota contributes to increased modulation of gut microbiome diversity and composition^37^. In this study, we concentrated on corroborating the association between hookworm infection, anthelminthic treatment, and the resulting effect on gut microbiome diversity. We found an association between hookworm infection and increased microbiota species richness. Studies across sub-Saharan Africa and parts of Asia have shown a similar pattern of association in both mixed and single-helminth infections^2,38,39^.

There was an increased relative abundance of the bacteria belonging to class *Clostridia* among infected individuals and an increase in both *Prevotella* and *Succinivibrio* among uninfected individuals. Commensal *Clostridia* are anaerobic gram-positive bacteria^40^. During active infection, *Clostridia* plays an essential role in gut defence mechanisms, both directly through colonisation resistance and indirectly through immune cell priming and modulation of immunological tolerance^41,42^. Studies comparing microbiome composition between rural and urban areas revealed an increased abundance of *Prevotella* and *Succinivibrio* in rural settlers^43,44^. *Prevotella* and *Succinivibrio* species are involved in polysaccharide metabolism and dietary fibre fermentation^44–46^. Their association with uninfected participants may occur because of an increased intake of plant-based diets^47^ in an agrarian lifestyle cultivated by people living in the study site, Kintampo North Municipality of Ghana^48^.

Our study also showed a significant variation in microbiota composition 10–14 days after single-dose albendazole treatment. Individuals who were successfully treated showed a significant reduction in microbiota composition when compared to their pre-treatment state, while individuals who failed to clear the helminth infection after treatment showed no significant change in microbiota composition. A similar study in Western Kenya also reported changes in the abundance of microbial taxa after successful helminth clearance post albendazole treatment^49^. This in line with our findings that successful single dose albendazole treatment may result in structural and compositional change to the gut microbiome. Notwithstanding this, it must be noted that, this alteration in microbiome structure and composition could be because of microbial rearrangement in response to adult worm and egg clearance and not a direct effect of drug administration^50^.

It is known that gut microbial signatures could be used to predict patient response to antibiotic therapy^51^. Microbiota composition was different between individuals who got cured and those that did not. The abundance of *Clostridia* was increased in positive individuals before treatment and continued to show an increased abundance in individuals who failed treatment. The gut microbiome of individuals who failed treatment also saw an enrichment of high-level pathways associated with pyruvate ferredoxin oxidoreductase (PFOR). PFOR catalyses the oxidative decarboxylation of pyruvate to carbon dioxide and acetyl-CoA^52^ and is a key enzyme in *Clostridia* metabolism as it is required to promote heterotrophic and lithoautotrophic growth in anaerobic bacteria^53^. The correlation between PFOR pathways and individuals who failed treatment could be associated with the increased abundance of strict anaerobes such as *Clostridia* spp. within the gut microbiome. These findings suggest persistent *Clostridia* as a potential predictive indicator for treatment failure in hookworm infection.

The gut microbiome has been demonstrated to undergo complete or partial reversion to its pre-treatment state in adults after cessation of anti-microbial therapy^54–56^. *Prevotella* was increased in individuals negative for hookworm infection and also after successful albendazole treatment. This increased representation of *Prevotella* in successfully treated individuals could point to a compositional shift in microbiota abundance towards a pre-infection state. However, when hookworm negative individuals were compared to individuals who successfully cleared the infection, the two groups were dissimilar. Whilst this dissimilarity implies a non-reversal of the microbiome structure after single dose albendazole therapy, we speculate that 10–14 days may not have been enough time for a significant reversion in microbiota composition to become apparent.

We recommend the application of meta-transcriptomics to ascertain microbial gene expression patterns^57^ in albendazole treatment failures to better understand the correlation between *Clostridia spp.* and treatment failure. Metabolomics can also be used to identify metabolites produced by *Clostridia spp.* and explore the possibility of using probiotic supplementation^58–60^ as an adjunct treatment in the bid to overcome treatment albendazole failure, especially when distributed in mass drug administration programs targeting hookworm infection.

Although studies have referred to bead associated mechanical lysis as the gold standard lysis technique for faecal DNA extraction^61^, newer studies have revealed that bead homogenization may lead to biases in characterising the microbiome^62–64^. As such, our choice to incorporate bead homogenisation within the study could serve as confounding variable. The sample size being small served as a limitation for our study. We will be performing follow up studies with significantly larger participant numbers to validate the results of this pilot study.

## Conclusions

This study focused on the role the gut microbiome plays, if any, in influencing cure rates after single-dose albendazole treatment. We found that the composition and diversity of the gut microbiota change in response to treatment. We also found *Clostridia* to be a potential microbial indicator of albendazole treatment failure. These data corroborate the concept that specific microbial taxa or taxa assemblages can be used as significant discriminants in the assessment of the effects of helminth infection and response to treatment on the human gut microbiome.

Our study contributes to the ongoing discussion on the effects of helminth infections and anthelminthic treatment on the human gut microbiome. With the reduction in the cost of 16S rRNA sequencing and the advent of open-source computational pipelines, we look forward to conducting subsequent well-regulated studies on the impact of gut microbiome changes on human health.

## Supplementary Information

Supplementary Information.

## Data Availability

All data generated from 16s gene amplicon sequencing are available in the NCBI Sequence Read Archive (SRA) via http://www.ncbi.nlm.nih.gov/bioproject/923309. All other original contributions presented in the study are included in the article, further inquiries can be directed to the corresponding author.
